# Clinical Outcomes of Tibial Components with Modular Stems Used in Primary TKA

**DOI:** 10.1155/2014/651279

**Published:** 2014-02-05

**Authors:** Nicole Durig, Thomas Pace, Brandon Broome, Obi Osuji, Melinda K. Harman

**Affiliations:** ^1^Department of Bioengineering, 301 Rhodes Engineering Research Center, Clemson University, Clemson, SC 29634, USA; ^2^University of South Carolina, School of Medicine Greenville, 607 Grove Road, Greenville, SC 29605, USA; ^3^Steadman Hawkins Clinic of the Carolinas, 200 Patewood Drive, Suite C100, Greenville, SC 29615, USA

## Abstract

Due to the known potential for fretting and corrosion at modular junctions in orthopaedic implants, this retrospective study evaluated radiographic and clinical outcomes of 85 primary TKA patients implanted with modular stemmed tibial components and followed up for an average of 82 months. There was low incidence of tibial radiolucent lines, excellent functional outcomes, and no complications associated with stem modularity. The findings were comparable to the historical control study involving 107 TKA with a nonmodular tibial stem design. When using surface cemented tibial components combined with a constrained polyethylene bearing, modular stems appear to be a viable option for primary TKA when adequate fixation and rotational stability are maintained.

## 1. Introduction 

Central stems on tibial components in total knee arthroplasty (TKA) can exist in many different lengths (short < 50 mm; long > 50 mm) and shapes (e.g., cruciform keels, I-beams, and cylinders). Whether provided in monoblock or modular form, central stems are thought to aid component fixation by transferring load-bearing stresses to stronger distal bone and by resisting component tilting and liftoff [1–7]. Some tibial component designs offer stem modularity, providing surgeons intraoperative flexibility to accommodate variable bone quality through attachment of differently shaped short modular stems for use in primary TKA [8–10] or long modular intramedullary stems when addressing bone deficiencies in revision knee arthroplasty [10, 11]. Modular stems typically are attached to the tibial baseplate using Morse tapers, with axial screws providing additional fixation in some designs.

Surgeons often restrict modular stem use to revision knee arthroplasty or to primary TKA cases with gross malalignment or osseous defects [12, 13]. During routine primary TKA, there are concerns that stemmed components restrict flexibility in baseplate positioning [1], contribute to reduced bone density in the tibial metaphysis [14], and may not significantly improve baseplate stability in all patients [6]. However, with use of more constrained polyethylene inserts during primary TKA, use of short modular stems to aid fixation can be rationalized since some studies predict an associated increase in interface stresses affecting fixation [10, 15, 16]. Selectivity also is driven by potential risks of stem modularity, including disengagement of the locking mechanism, dissociation and micromotion at the modular junction, and increased potential for generating metal ions and debris due to fretting wear or corrosion [17–24]. Consequently, clinical outcomes for short modular stems routinely used in primary TKA are infrequently reported [8, 9]. Given recent heightened clinical concerns related to modularity and the potential for adverse tissue responses to metal debris [19, 20, 23–28], ongoing vigilance of primary TKA having tibial components with modular stems is warranted.

The aim of this study is to evaluate clinical outcomes at the 2-to-11-year follow-up interval for a consecutive series of primary TKA patients implanted using a single prosthesis design having a cemented tibial baseplate with five pegs and a short modular stem attached via a Morse taper and a conforming polyethylene insert. The rationale for selecting this prosthesis design for primary TKA was to provide anteroposterior stability at the femoral-tibial articulation, rotational stability from the pegs at the baseplate-bone interface, and varus-valgus stability from the stem to prevent baseplate liftoff. Because this study is focused on primary TKA, we identified a historical control study inclusive of a comparable TKA design with similar tibial component features, except for the stem modularity [29]. The null hypothesis was that two cohorts of cemented tibial components with modular stems and nonmodular stems would have equivocal outcomes. Due to the known potential for fretting and corrosion at modular junctions, the alternate hypothesis was that these primary TKA patients with modular stems would have greater radiographic evidence of tissue reactions and complications associated with modularity, with higher revision rates compared to historical controls without modularity.

## 2. Materials and Methods

Between May 2001 and October 2002, 121 consecutive knees in 119 patients underwent primary TKA by a single surgeon and coauthor Thomas Pace. While all data points were entered prospectively concurrent with each patient's pre- and postoperative clinical assessment, the results were retrospectively reviewed in this institutional review board-approved study. Only patients who achieved a minimum of 2 years of follow-up were evaluated, resulting in exclusion of 24 patients (25 TKA). Two patients (2 TKA) were lost to follow-up and 9 patients (9 TKA) died before the 2-year follow-up interval. The final cohort consisted of 85 TKA in 84 patients with 2 to 11 years of follow-up. There were 60 females and 24 males, with an average age at index surgery of 66 ± 11 (31–86) years.

**Figure 1:**
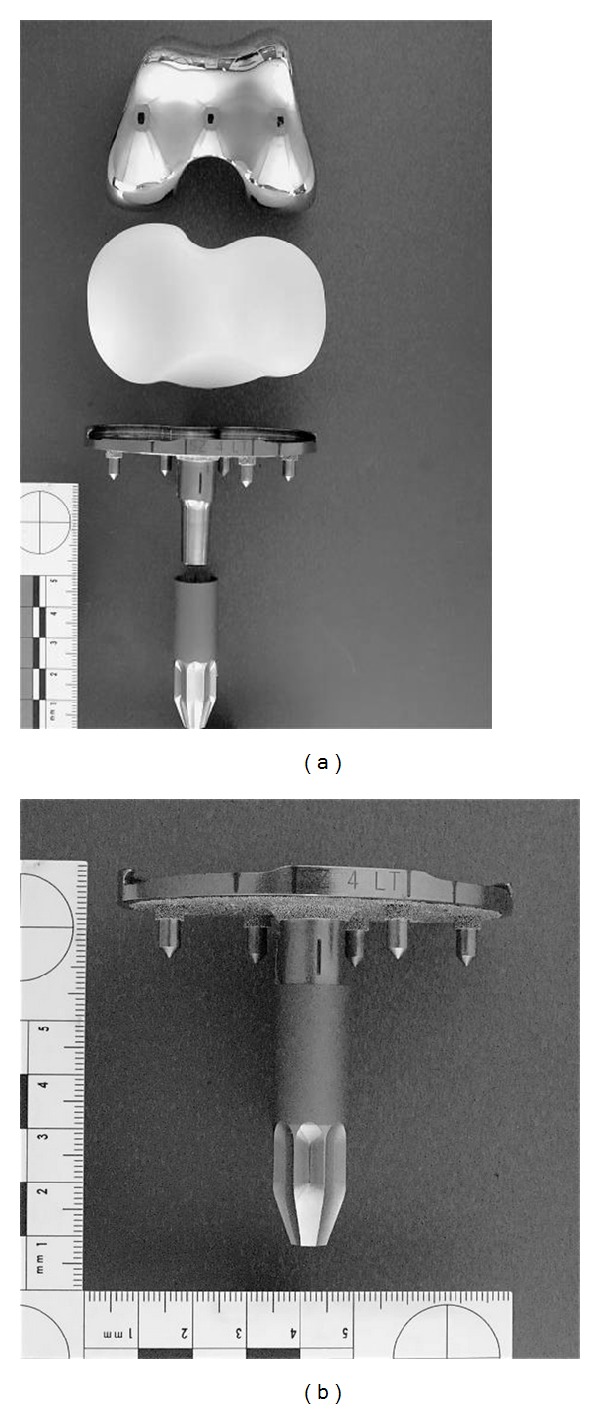
Gross photographs of disassembled individual components of the Profix TKA (a) and an assembled tibial component with stem (b) used in the current study.

All patients were implanted with the same prosthesis design (Profix Total Knee System, Smith & Nephew, Memphis, TN) that incorporates a modular stem attached to the tibial baseplate (Figure 1). The tibial component was fabricated from titanium alloy and the geometry consisted of an asymmetric baseplate with a porous distal surface, 5 peripheral pegs, and a surface-textured modular central stem. This design provides the option for using one of four different central stems; however, all patients in this study received the same short, surface-textured stem (Figure 1). Tibial components were fixed using a surface cementation technique with bone cement (Palacos R cement, Biomet Inc., Warsaw, IN) applied to the undersurface of the baseplate, excluding the pegs and stem. The femoral components were fabricated from oxidized zirconium (Oxinium, Smith & Nephew, Memphis, TN) in 15 knees and cobalt-chrome alloy in 70 knees. All tibial inserts were machined from conventional (not highly cross-linked) ultrahigh molecular weight polyethylene (UHMWPE) and had been sterilized in ethylene oxide. All knees were implanted with an insert having anterior constraint (Conforming Plus, Smith & Nephew, Memphis, TN).

**Figure 2:**
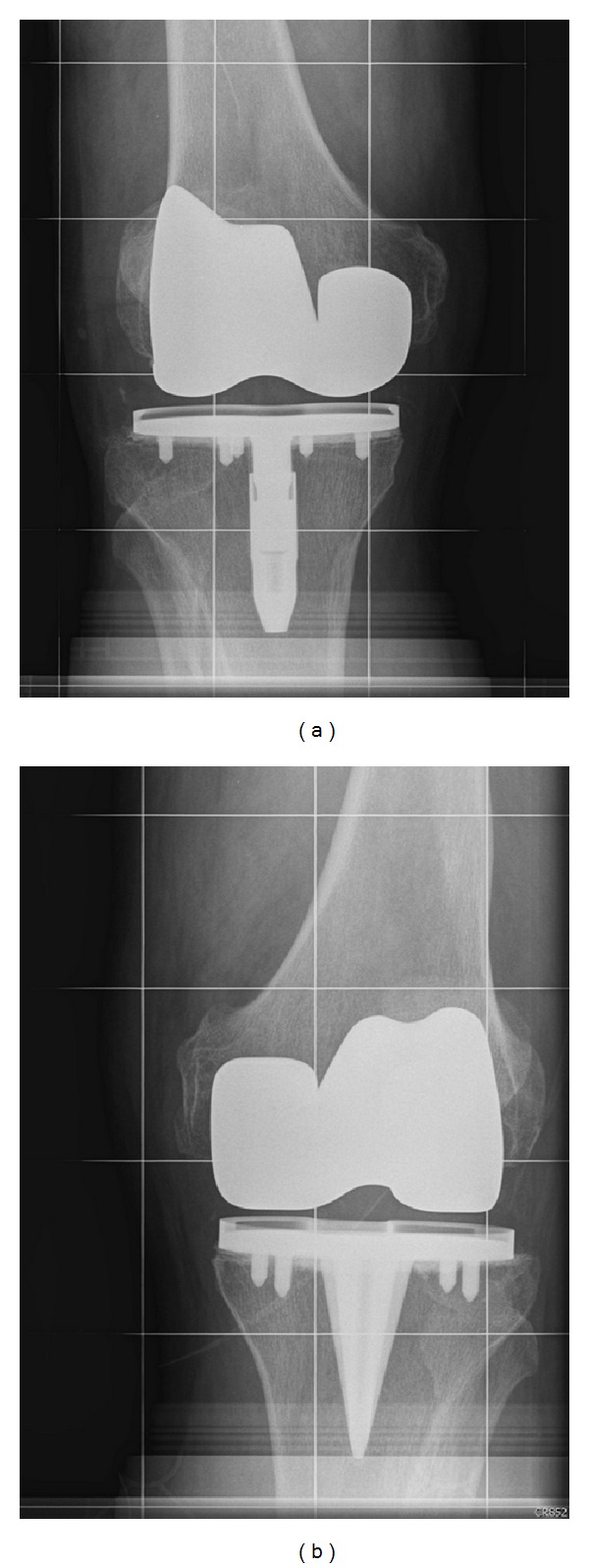
Anteroposterior radiograph of a bilateral TKA patient implanted with (a) the Profix Total Knee System (right knee) and (b) Natural Knee II (left knee) used in the historical control study.

Medical records were retrospectively reviewed to assess clinical and radiographic outcomes at last the follow-up. The surgical approach and posterior cruciate ligament treatment (retention or sacrificed) were recorded, as well as preoperative and postoperative Knee Society Scores [30] and postoperative range of motion (ROM). Any noted complications or subsequent revision surgeries were noted. Radiographic analysis consisted of two independent observers assessing full-length, standing anteroposterior, sunrise, and lateral views (Figure 2) and recording the presence of osteolytic lesions and any radiolucent lines greater than 2 mm that were located under the surface-cemented tibial baseplate. Implant failure was defined as tibial osteolysis and/or progressive tibial baseplate radiolucency per serial radiographs, revision knee surgery for implant-related problems, or significant leg pain likely attributable to insufficient tibial implant fixation.

### 2.1. Historical Control Study

This level III therapeutic study, including patients treated with a primary TKA having a modular tibial stem, was compared to a historical control group of patients treated with a primary TKA having a nonmodular tibial stem, as reported by Hofmann et al. [29]. This prior study was chosen because the TKA prosthesis evaluated (Natural Knee II, Zimmer, Warsaw, IN) is similar to the design used in the present study. Similarities include the use of an asymmetric titanium tibial baseplate having a smooth central stem and peripheral pegs, with the baseplate fixed using a surface cementation technique with bone cement (Simplex P cement, Howmedica, Rutherford, NJ) impregnated with 1.2 g of tobramycin per 40 g of cement applied to the undersurface of the baseplate, excluding the pegs and stem. The main difference in design is the use of a fixed stem in the historical control study and a modular stem in the present study.

Hofmann et al. [29] reported a retrospective review of 128 consecutive primaries TKA implanted between 1991 and 1998, with 107 knees in 88 patients available for review at a minimum of 5-year follow-up. Eighteen males and seventy females were included with an average age of 74 (range 46–91) years. Recorded data included diagnosis, surgical technique, and posterior cruciate ligament treatment, as well as preoperative and postoperative range of motion (ROM), modified Hospital of Special Surgery (HSS) Score, Knee Society Score, and radiographic findings. Radiographic data included alignment, radiolucent lines, and osteolytic lesions (defined as an expanding area of focal radiolucency of at least 1 cm) evaluated on full-length standing, anteroposterior, lateral, and sunrise views.

## 3. Results

Indication for TKA for all patients was osteoarthritis. Patients with other indications (e.g., rheumatoid arthritis) received a different TKA design and were not included. The posterior cruciate ligament (PCL) was sacrificed in all 85 TKA, with anteroposterior stability provided through the use of a polyethylene insert incorporating an anterior-constrained articular geometry. The patella was unresurfaced in all but one knee. The medial parapatellar approach was used in 83 (97.6%) of the cases, whereas the remaining 2 (2.4%) were exposed using the subvastus approach. The subvastus approach was selected on a case by case basis with the premise that it may offer some benefit by allowing a quicker recovery and less postoperative pain. It was performed on patients if the distal thigh circumference was small enough that it could practically be done; however, patients with a larger distal thigh circumference received the medial parapatellar approach.

The average follow-up was 82 ± 38 (24–133) months, including 56 TKA with a minimum of 5-year follow-up. Average preoperative Knee Society Scores were 78.6 ± 4.6 (70–87) and improved to 99.2 ± 2.0 (90–100) postoperatively. Postoperative flexion averaged 118.5° ± 5.4° (95°–128°).

No radiographs demonstrated osteolytic lesions around the tibial component. Radiolucent lines adjacent to 2 TKA were noted upon initial radiographic analysis. In one TKA, the radiolucent line was less than 2 mm in thickness, asymptomatic, and not associated with prosthesis failure. In the other TKA, a 2 mm lucent line was noted and further investigated. The patient had indicated mild patellofemoral pain (not requiring medication), most likely related to the unresurfaced patella and is not considered to be associated with failure of the tibial component.

None of the knees included in the current study required revision surgery. No infections were recorded in this series of patients. One knee experienced dehiscence two weeks following surgery and was treated with secondary wound closure and healed uneventfully. One knee required closed, manual manipulation following implantation because the average flexion was less than 90° at the 10–12 week follow-up visit. The overall survivorship was 100% at an average of 82 months with no pending failures.

### 3.1. Historical Control Study

The historical control study by Hofmann et al. [29] included patients treated for primary osteoarthritis (76 patients), rheumatoid arthritis (10 patients), and posttraumatic arthritis (2 patients). The PCL was retained in 35 TKA and sacrificed in 53 TKA, with subsequent anteroposterior stabilization provided by the use of a polyethylene tibial insert incorporating an anterior-constrained articular geometry (Ultracongruent, Zimmer, Warsaw, IN). The subvastus approach was used in 67 knees, while 21 knees received the medial parapatellar approach.

Average follow-up for the control group study was 95 (range 63–155) months. Postoperative Knee Society Scores averaged 195 (range 162–200), which improved from the preoperative scores that averaged 122 (range 94–152). Postoperative flexion averaged 116°.

Radiographic review of the historical control group revealed no osteolytic lesions in any TKA. However, three TKA had nonprogressive radiolucent lines adjacent to the tibial baseplate, which all were asymptomatic and not associated with prosthesis failure. Two other TKA required revision surgery but none of the tibial components were revised for loosening. One revision consisted of a polyethylene exchange due to PCL insufficiency and the other required a femoral component removal due to pain and possible loosening at 4 years following index surgery. There were no infections reported. The overall survivorship was 98% at an average of 95 months.

## 4. Discussion

The recorded positive clinical outcomes, including lack of radiolucent lines and osteolysis in these primary TKA with surface-cemented tibial components and short modular stems, indicate that fretting and corrosion were not prominent features of the modular junction at the 2-to-11- year follow-up interval. The lack of revisions, few complications, low incidence of tibial radiolucent lines, and excellent functional outcomes were comparable to the historical control study involving patients implanted with pegged tibial baseplates having a nonmodular central stem [29]. The early to mid-term outcomes in the current study are comparable to other recent studies using the same modular tibial stem prosthesis design [8, 31]. Hardeman et al. [8] reported greater than 97% survivorship at 10 years after cementless TKA. In a recent literature review including a pooled cohort of 1152 TKA, Viganò et al. [31] reported Knee Society Scores greater than 90 and 10-year survivorship of 94.2% and 100% with endpoints of revision for any reason and for radiographic loosening, respectively.

There are several factors that likely contributed to our results. First, tibial stems were used during primary TKA in the absence of large tibial bone defects. In such circumstances, stemmed components endure lower load magnitudes and decreased stresses, resulting in greater baseplate stability than the same components implanted in tibias with bone defects [32]. In the current study, patients with rheumatoid arthritis received a different prosthesis design. In our patient cohort of osteoarthritic patients, there was likely sufficient dense bone able to provide adequate support to the short modular stems [5, 6]. Second, tibial baseplates with pegs are rotationally stable, which helps to reduce the torsional stresses transmitted to the modular interface. Good clinical outcomes have been reported for TKA designs having such augmented fixation [29, 33–36]. Third, the surface cementing technique leaves the modular stem uncemented, which has been shown to maintain a more even proximal bone stress distribution and may have shielded the modular junction from torsional loads generated at the constrained articular surface [37, 38]. Finally, all tibial baseplates and stems were fabricated from titanium alloy materials, eliminating the potential for corrosion-related complications known to occur with modular couples comprised of dissimilar metals [23, 39, 40].

The surgical technique of the senior surgeon used in these PCL-resected primary TKA included routine selection of a conforming polyethylene insert with anterior constraint rather than a cam and post-PCL-substituting (PS) design and implantation of a modular stem tibial baseplate to augment tibial baseplate. Several clinical studies support this surgical technique. Hofmann et al. [35] report similar clinical outcomes when using either PS or ultracongruent designs during primary TKR. In 231 TKA operated with a PCL resecting technique, Straw et al. [41] showed that it is not essential to use a PS insert as long as reasonably conforming inserts are used. Other studies report no substantial differences in clinical outcomes, improvement in flexion range, or stair climbing abilities in primary PCL-resected patients receiving a deep-dish or PS TKA design [42, 43]. Laskin and Davis [42] conclude that using a deep-dish insert obviates the need to resect intercondylar femoral bone, decreasing the potential for fracture and maximizing bone volume should revision be necessary in the future.

It was not the intent of this study to compare complication rates for modular tibial stems used in primary and revision TKA, as considerable differences exist in those different clinical scenarios [10]. Although few, case reports of all taper disassembly or corrosion involve complex revision TKA [17, 18, 21–23]. In the presence of bone defects, long intramedullary stems carry a considerable proportion of the axial loads [32, 44], but the relationship between in vivo loading conditions and taper complications in TKA is unclear. Nevertheless, vigilance of modular TKA used in primary and revision clinical scenarios is warranted because corrosion can occur even in well-fixed TKA components. McMaster and Patel [23] report corrosion adjacent to a femoral modular stem within 2 years of revision TKA for a medial femoral condylar fracture. Radiographs showed no change in component orientation, radiolucent lines, or osteolysis. Intraoperatively, the authors report a bloom of black encrustations at the Morse taper junction of the distal femoral component and its well-ingrown porous-coated intramedullary femoral stem extension.

There are some limitations to this study. The average follow-up time of approximately seven years may not be sufficient to fully capture potential adverse events. However, particulate wear-induced osteolysis can present radiographically within the first three years [45]. Therefore, patients with a minimum of two years of follow-up were included in the current cohort. Additionally, the number of included patients may not be sufficient to appreciate a potentially very small incidence of adverse events. This study includes no intrinsic control group; rather, it uses a historical control study [29] that we believe is sufficiently comparable because of the similar patient cohort and TKA design, differing primarily in tibial stem modularity.

In these primary TKA patients implanted with surface cemented tibial components having a short modular stem and constrained polyethylene bearing, there were no complications associated with stem modularity. The absence of progressive radiolucent lines is consistent with adequate fixation, including rotational stability provided by the pegs and sufficient varus-valgus stability provided by the modular stem. These findings do not support the null hypothesis that these modular TKA experience greater radiographic evidence of negative tissue reactions and higher revision rates compared to patients receiving TKA without stem modularity. At this 2–11-(average 6.8) year follow-up interval, the option for attaching modular stems appears to be a viable design feature for primary TKA. However, since only one modular stem TKA design was evaluated after used with uniform surgical techniques during routine primary TKA, the authors caution that these findings may not be applicable when generalized to other designs with different modular stems and articular conformity used under different clinical scenarios.
